# Adenocarcinoma of the gallbladder: an incidental histopathological finding post-operative laparoscopic cholecystectomy in Saudi Arabia

**DOI:** 10.25122/jml-2025-0095

**Published:** 2025-10

**Authors:** Ahmed Mihdhar Saggaf, Ali Mohamed Nagi, Ranim Mustafa Al Atat, Mihdhar Omar Saggaf, Jumana Hussain Timraz, Husna Irfan Thalib, Lujain Mohammed Alkhateeb

**Affiliations:** 1 Department of General Surgery, King Abdulaziz University Hospital, Jeddah, Saudi Arabia; 2 Prince Meshari Bin Saud General Hospital, Al Baha, Saudi Arabia; 3 Ibn Sina National College Faculty of Medicine, Jeddah, Saudi Arabia; 4 Batterjee Medical College for Sciences and Technology, Jeddah, Saudi Arabia; 5 Dr. Solaiman Fakeeh Hospital, Jeddah, Saudi Arabia

**Keywords:** gallbladder, gallbladder cancer, adenocarcinoma, laparoscopic cholecystectomy, incidental histopathology

## Abstract

Gallbladder adenocarcinoma (GBC) is a rare and challenging diagnosis, often associated with chronic gallstones and discovered incidentally during histopathological examination. It may present in early or late stages without overt clinical or radiological signs. Laparoscopic cholecystectomy is commonly performed for gallstones, and in such cases, gallbladder cancer may be detected even in the absence of a visible mass or atypical clinical presentation. A 68-year-old female with no significant medical history presented with a gallstone, confirmed via abdominal ultrasound, showing multiple stones and sludge. The patient underwent an elective laparoscopic cholecystectomy without intraoperative complications, such as bile spillage, significant bleeding, or organ injury. Postoperative recovery was uneventful, with no additional symptoms. Histopathological analysis revealed grade 2 (moderately differentiated) adenocarcinoma extending to the hepatic surface of the gallbladder. Gallbladder adenocarcinoma can present silently in its early stages, underscoring the importance of histopathological examination of all cholecystectomy specimens, especially in older patients with gallstones. Avoidance of bile spillage during surgery is crucial for improving prognosis. Further metastatic evaluation through chest, abdominal, and pelvic CT shows no abnormalities in this case. Regular follow-up with hepatobiliary and oncology teams was recommended.

## Introduction

Gallbladder carcinoma (GBC) is the most common malignancy of the biliary tract, with significant variation in incidence across ethnic and geographic groups. It is considered an aggressive tumor, with approximately half of cases being diagnosed incidentally at advanced stages [1]. In Saudi Arabia, 1,678 cases of GBC were reported between 2004 and 2015, with females accounting for 976 cases and males for 702. The majority of patients were aged 75 years or older, placing them at higher risk for GBC [2].

The primary risk factor for developing GBC is the presence of gallstones and biliary duct stones, which are strongly associated with chronic gallbladder inflammation. This chronic inflammation promotes metaplasia, dysplasia, and eventual carcinoma [2]. Additional risk factors include female gender, advanced age, elevated body mass index (BMI), low high-density lipoprotein cholesterol, and gallbladder polyps [3].

A study found that multiple small gallstones (<1.0 cm) were present in 57.9% of patients with GBC, with cholesterol stones being the most common. Interestingly, differences in gallstone composition among Saudi patients highlight a need for further regional studies [4]. Obesity, with a BMI ranging from 31.0 to 40.0 kg/m^2^, and diabetes mellitus are also strongly linked to GBC. The underlying mechanisms remain unclear, but it is hypothesized that obesity leads to endocrine dysfunction, increasing the risk of GBC. The variability in the ethnic and geographic prevalence of obesity may contribute to differences in GBC incidence patterns. However, more multi-center and national studies are required to understand better these associations in the Saudi population [4].

This report presents an incidental finding of gallbladder adenocarcinoma diagnosed postoperatively following laparoscopic cholecystectomy in Saudi Arabia. The patient displayed no clinical, biochemical, or radiological features indicative of malignancy pre-operatively.

## Case presentation

A 68-year-old female with no significant comorbidities and no family history of gallbladder cancer or other malignancies was admitted for elective laparoscopic cholecystectomy. The patient presented without any symptoms typically associated with gallbladder tumors, such as weight loss, loss of appetite, abdominal pain, nausea, vomiting, anorexia, fever, or dark-colored urine. There were also no signs of advanced metastasis, such as obstructive symptoms.

On clinical examination, no palpable lump was detected in the right upper quadrant, and there were no signs of jaundice or pruritus. The presentation was atypical for a gallbladder tumor, and no abnormalities were noted radiologically or intra-operatively. Pre-operative biochemical markers, including alpha-fetoprotein, CA 125, CA 15-3, CA19.9, and carcinoembryonic antigen (CEA), were all within normal limits.

**Figure 1:**
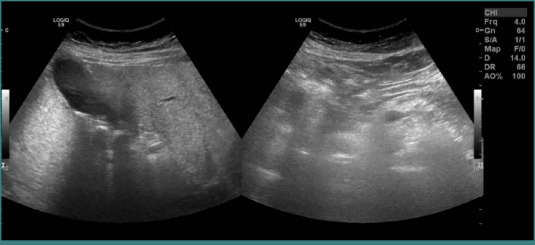
An abdominal ultrasound (US abdomen). The gallbladder contains multiple stones and sludge without mass as a pre-operative assessment.

**Figure 2:**
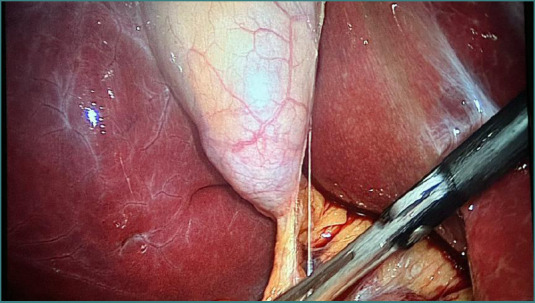
Intraoperative laparoscopic image showing the gallbladder during dissection

An abdominal ultrasound revealed multiple gallstones and sludge but showed no masses or anatomical abnormalities (Figure 1). The laparoscopic cholecystectomy was performed without intraoperative complications, including bleeding, bile spillage, or injury to adjacent structures (Figure 2). As per the hospital’s protocol, the gallbladder specimen was sent for routine histopathological examination.

**Figure 4:**
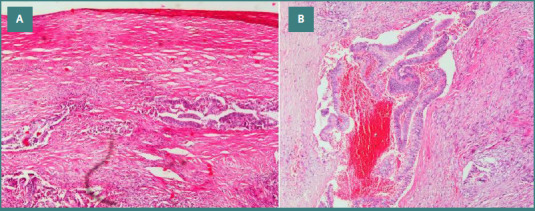
A, A histopathology examination of the gallbladder with hyalinization and embedded malignant glands (H&E, 200x). B, Higher magnification of the malignant glands and surrounding stroma with occasional inflammation (H&E, 400x).

**Figure 3:**
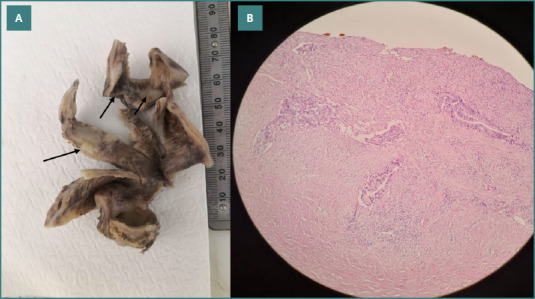
A, Gross histopathology examination of the gallbladder showing a mildly thickened area (arrows), though no obvious papillary or polypoidal mass is grossly visible. B, Microscopic features showing malignant glands invading the muscle layer with vascular permeation (H&E, 100x).

Gross and microscopic examination of the specimen revealed a tumor located in the body and neck of the gallbladder, with the largest dimension measuring 3.5 cm and a thickness of 0.7 cm (Figure 3A). Microscopic evaluation identified a grade 2 (moderately differentiated) adenocarcinoma. The tumor invaded the muscle layer and connective tissue, extending to a rough area on the hepatic surface. No liver tissue was included in the specimen, but vascular invasion was observed (Figure 3B). Although the lesion was sizable, the pre-operative ultrasound was carefully re-reviewed following the histopathological diagnosis; however, no distinct mass or abnormality was retrospectively appreciated. Pathology clarified that the lesion had a flat, infiltrative morphology, which explains why it was radiologically occult despite its size. Microscopic examination revealed malignant glands invading the muscle layer and extending into the connective tissue toward the hepatic surface, accompanied by areas of hyalinization and a surrounding stromal inflammatory response (Figure 4AB).

Post-operative assessment included chest, abdominal, and pelvic CT scans with double contrast to evaluate for metastasis. These studies revealed no evidence of metastatic disease or suspicious masses.

The patient remained stable throughout the post-operative recovery phase, with no complications reported during the hospital stay. The case was referred to a hepatobiliary and oncology surgery team for further management. Follow-up was scheduled every 6 months to monitor for recurrence or progression and to ensure appropriate oncological care if indicated.

## Discussion

Incidental gallbladder carcinoma refers to malignancy detected for the first time during routine histopathological examination of gallbladder specimens following cholecystectomy [5]. GBC is an aggressive malignancy characterized by frequent local invasion and distant metastases. In the United States, more than 1,000 new cases of GBC are diagnosed annually, with 70% of these cases occurring in women. Incidence rates vary significantly by ethnicity, with rates of 64% among Caucasians, 17% among Hispanics, 9% among African Americans, and 2% among Pacific Islanders/Asians [6-8]. While a family history of gallbladder cancer is uncommon, other risk factors play a crucial role, including the presence of gallstones, prolonged inflammation, advanced age, and female gender. In this case, the combination of gallstones and advanced age likely contributed to the development of GBC. These findings reinforce the importance of routine histological examination of gallbladder specimens, particularly in high-risk groups [9]. GBC is notoriously difficult to diagnose due to its silent nature. Symptoms such as fever, abdominal pain, and jaundice are often mistaken for other conditions like pancreatitis, biliary stones, or hepatitis [9]. Histological examination is therefore considered the gold standard for detecting malignancy in such cases.

A seven-year retrospective study conducted in Qatar by Sulieman *et al*. identified incidental GBC in 40% of patients (14 out of 35) during routine histological examination. These patients had significantly better survival rates compared to those diagnosed based on clinical symptoms (100% vs. 77.8%, *P* = 0.02) [10]. There are several possible outcomes for incidental GBC detected postoperatively: (1) early-stage carcinoma cured by simple cholecystectomy, (2) incomplete resection during cholecystectomy, requiring subsequent radical re-resection, and (3) dissemination of malignancy due to intraoperative bile spillage, resulting in poor outcomes [11]. In this case, the tumor was detected at an early stage (likely T1b or T2), which is associated with a better prognosis. Importantly, there was no bile spillage during surgery, a known factor that worsens survival. Studies have demonstrated that bile spillage correlates with poorly differentiated tumors and is associated with significantly reduced outcomes [11].

Other studies have also emphasized the importance of routine histopathological examination in identifying incidental gallbladder cancer. Zhang *et al*. reviewed 10,466 laparoscopic cholecystectomy specimens and found a 0.19% incidence of incidental carcinoma, mostly in early stages, which were managed effectively with simple cholecystectomy [12]. Similarly, Geramizadeh *et al*. in 2017 reviewed 1,800 cholecystectomy specimens and found 18 cases of incidental gallbladder adenocarcinoma. Their study reported that most incidental cases were at an early stage (T1 or T2), and those diagnosed at the T1 stage had a favorable prognosis with simple cholecystectomy. In contrast, T2 cases required extended surgery, but their prognosis remained poor [13]. These findings support the routine submission of gallbladder specimens for histopathological analysis, especially in older patients and those with risk factors.

A study in Saudi Arabia by Aldossary *et al*. reviewed 76 patients with GBC. Only 3.9% (3 out of 76) were diagnosed postoperatively through routine histological examination. The majority presented at advanced stages (50% at Stage II and 50% at Stage IVB). Patients with Stage I GBC had a significantly higher survival rate, while those with advanced-stage disease frequently succumbed within one year. The overall median survival for advanced-stage patients was immeasurable due to clustering at low survival rates. However, patients who underwent surgical resection had a higher survival rate (58.9%, CI: 35.2%–76.5%, *n* = 25), compared to 0% for those with advanced metastases (*n* = 39). Only 5% of patients achieved a five-year survival rate [4].

In our case, the patient underwent a successful laparoscopic cholecystectomy without bile spillage or complications. Histopathological analysis revealed grade 2 adenocarcinoma. Risk factors such as advanced age and female gender likely contributed to the malignancy, but the absence of bile spillage and the early-stage diagnosis significantly improved the prognosis. These findings align with studies by Sulieman *et al*. and Aldossary *et al*., which highlight the survival benefits of incidental GBC detection [4,10].

When evaluating the case, it is essential to recognize certain limitations inherent to the available data. The quality and detail of the gross and microscopic specimen images were restricted by the retrospective design and institutional archival constraints, which precluded access to higher-resolution or additional materials. Despite these constraints, the findings remain clinically relevant and provide meaningful insight into the case.

While gallbladder adenocarcinoma is not uncommon globally, the incidental detection of early-stage disease in asymptomatic patients remains clinically significant. This case demonstrates several noteworthy aspects: (1) the tumor was identified in a patient with no clinical, biochemical, or radiological suspicion of malignancy, (2) histopathological examination revealed hepatic surface extension and vascular invasion despite the absence of intraoperative findings, and (3) the diagnosis was made within a demographic (Saudi Arabian female) where such incidental discoveries are particularly underreported.

## Conclusion

Gallbladder cancer can present silently in its early stages, making routine histopathological examination of gallbladder specimens critical, particularly in high-risk patients such as older females and those with a history of chronic cholecystitis. Early detection through histopathology provides a significantly better prognosis compared to biochemical or radiological testing alone. Surgeons should exercise caution during laparoscopic cholecystectomy to avoid bile spillage, as it is associated with worse outcomes. Postoperative management should include a thorough radiological oncology assessment using chest, abdominal, and pelvic CT scans with double contrast to rule out metastasis. Regular follow-up with hepatobiliary and oncology specialists is essential for ongoing surveillance and timely intervention if needed. Routine histopathological examination not only improves early detection rates but also increases survival outcomes, underscoring its importance in all cholecystectomy cases, regardless of clinical suspicion for malignancy. While gallbladder adenocarcinoma is not rare globally, the incidental detection of early-stage disease in asymptomatic patients remains clinically significant.

## Data Availability

All data generated or analyzed during this study are included in this published article.
